# Migrant and native women’s perceptions of prenatal care communication quality: the role of host-country language proficiency

**DOI:** 10.1186/s12889-023-15154-4

**Published:** 2023-02-09

**Authors:** Sousan Hamwi, Elsa Lorthe, Milton Severo, Henrique Barros

**Affiliations:** 1 EPIUnit– Instituto de Saúde Pública, Universidade Do Porto, Porto, Portugal; 2 Laboratório Para a Investigação Integrativa E Translacional Em Saúde Populacional (ITR), Porto, Portugal; 3 Unit of Population Epidemiology, Department of Primary Care Medicine, Geneva University Hospitals, Geneva, Switzerland; 4 Université Paris Cité, INSERM, INRA, Centre for Research in Epidemiology and Statistics Paris (CRESS), Paris, France; 5 Departamento de Ciências da Saúde Pública E Forenses, e Educação Médica, Faculdade de Medicina, Universidade Do Porto, Porto, Portugal

**Keywords:** Migrants, Maternal health services, Communication barriers, Physician–patient relation

## Abstract

**Background:**

Despite the potentially significant impact of women-prenatal care provider communication quality (WPCQ) on women’s perinatal health, evidence on the determinants of those perceptions is still lacking, particularly among migrant women.

**Methods:**

We aimed to examine the effect of women’s host-country language proficiency on their perceived WPCQ. We analyzed the data of 1210 migrant and 1400 native women who gave birth at Portuguese public hospitals between 2017 and 2019 and participated in the baMBINO cohort study. Migrants’ language proficiency was self-rated. Perceived WPCQ was measured as a composite score of 9 different aspects of self-reported communication quality and ranged from 0 (optimal) to 27.

**Results:**

A high percentage of women (29%) rated communication quality as “optimal”. Zero-inflated regression models were fitted to estimate the association between language proficiency and perceived WPCQ. Women with full (aIRR 1.35; 95% CI 1.22,1.50), intermediate (aIRR 1.41; 95% CI 1.23,1.61), and limited (aIRR 1.72; 95% CI 1.45,2.05) language proficiencies were increasingly more likely to have lower WPCQ when compared to natives.

**Conclusions:**

Facilitating communication with migrant women experiencing language barriers in prenatal care could provide an important contribution to improving prenatal care quality and addressing potential subsequent disparities in perinatal health outcomes.

**Supplementary Information:**

The online version contains supplementary material available at 10.1186/s12889-023-15154-4.

## Introduction

A large and growing body of literature has recognized the pivotal role of utilization adequacy and quality of prenatal care in maternal and infant health, as well as family wellbeing as a whole [1, 2]. Prenatal care typically involves the vital tasks of pregnancy risk assessment; prevention, management, treatment of pregnancy complications or comorbidities; and health education and promotion—all carried out by skilled healthcare professionals [1].

According to the World Health Organization, establishing effective communication between pregnant women and their prenatal health care providers is a key element of prenatal care quality [1, 2]. Effective communication is a two-way verbal and non-verbal information exchange that seeks to convey relevant knowledge, involve women in decision-making, and create a good interpersonal relationship between women and their care providers [1, 3]. For women-prenatal care provider communication to be effective, both parties should be able to listen and speak without interruptions, feel free to ask questions, express opinions and worries, and fully understand one another [4]. Women’s perceptions of communication quality during prenatal encounters could directly influence their perinatal health decisions and outcomes [5]. Studies of perceived patient-provider communication quality have shown a link with a wide range of important and interrelated health outcomes including health literacy, utilization and satisfaction with care, adherence to recommendations and treatments, future healthcare use and engagement in decision-making, and, consequently, quality of life and emotional and physical health [6–9].

Patient-provider communication quality can be influenced by patient factors such as age, gender, ethnicity/race, socioeconomic status, health literacy, self-efficacy, and perceived general health, and healthcare factors such as quality and content of care, duration of consultations, attitudes of health providers, and continuity and trust in the relationship with a provider [10–12]. Migration background has also been linked to the perceived quality of patient-provider communication [12–14]. In the context of perinatal health, a recent review on involvement in maternal care by migrants and ethnic minorities concluded that migrants are less likely to understand healthcare professionals, obtain adequate information, express or have their preferences granted, and be involved in decisions about their perinatal health when compared to natives [15]. Time constraints, language barriers, lack of adequate interpreting services, socio-cultural beliefs that discouraged active participation in decision-making, and attitudes of care providers all contributed to migrants’ lack of involvement in maternity care [15]. However, most of the evidence regarding these explanations came from qualitative studies.

In the current study, we chose to focus on the role of host-country language proficiency in perceived communication quality, since language difficulties are among the most frequently documented barriers to the access and quality of prenatal care. More specifically, we aimed to examine the effect of women’s host-country language proficiency on their perceived women-prenatal care provider communication quality (WPCQ). We hypothesized that lower language competencies would result in worse perceptions of WPCQ.

## Materials and methods

### Study design and participants

This study draws on cross-sectional data collected as part of the baMBINO cohort (Perinatal Health in Migrants: Barriers, Incentives, and Outcomes)—a Portuguese nationwide study led by our team in the Institute of Public Health of the University of Porto and designed to investigate migrant women’s perinatal healthcare experiences and outcomes compared to native women [16, 17].

All 39 Portuguese public hospitals with maternity units were invited to collaborate, and 32 (82%) accepted. In 2018, the collaborating units accounted for almost 85% of total deliveries in mainland Portugal [18]. Between April 2017 and March 2019, all native and migrant women who were at least 18 years old and had a live birth in one of the collaborating maternity units were considered eligible for participation in baMBINO. Migrant women were defined as those born outside of Portugal and were invited to participate in the study during their delivery hospital stay. For each migrant woman who consented to participate, the following native woman giving birth at the same hospital was also invited.

**Fig. 1:**
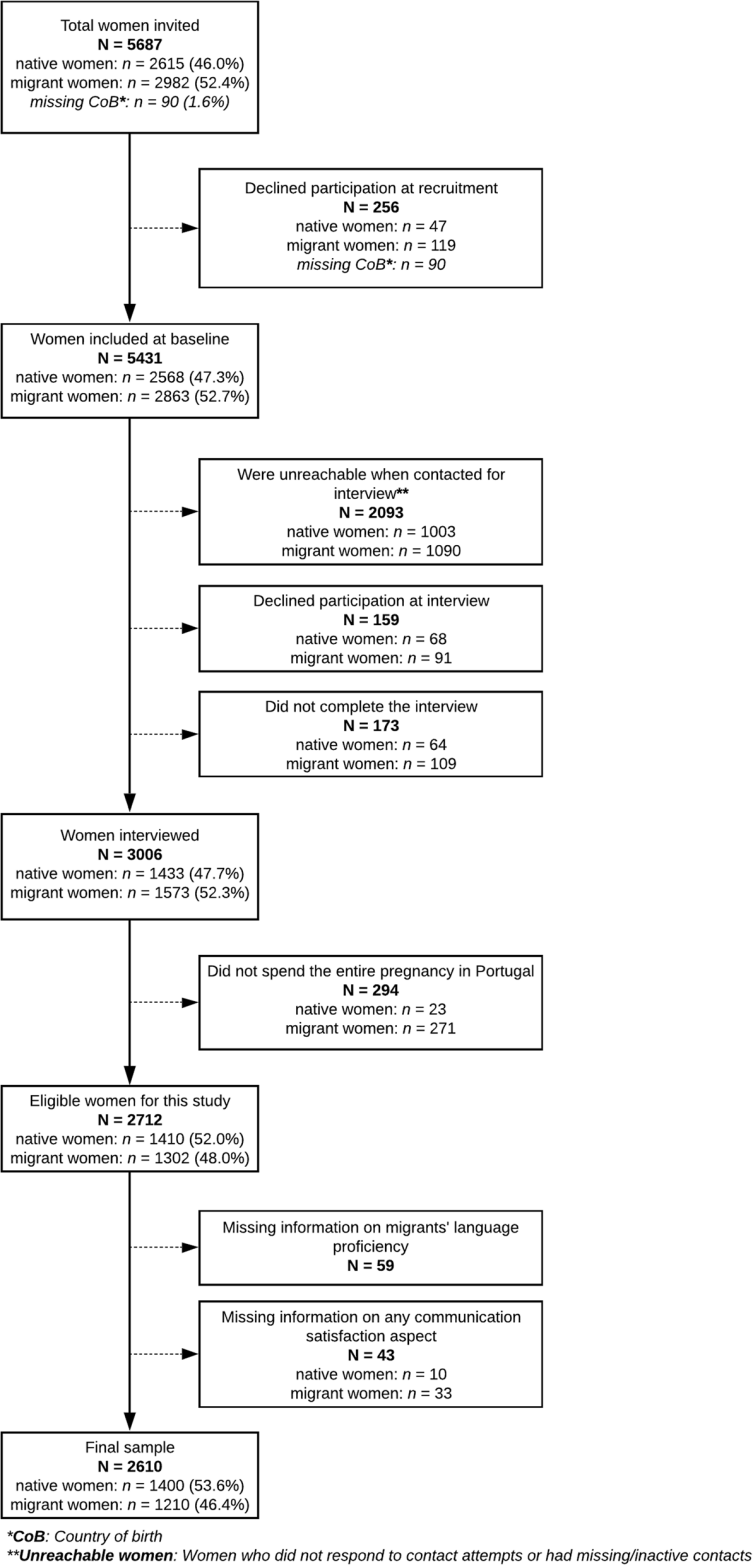
Flowchart of participants

Out of 5687 women invited, 5431 (95.5%) consented to participate, of which 2863 (52.7%) were migrants (Fig. 1). Medical staff on duty collected the sociodemographic and clinical data of included women using their electronic medical records. Participants were contacted after hospital discharge by a team of trained multi-lingual interviewers to complete a computer-assisted telephone interview. Women were interviewed in their language of choice, using professional interpreters when needed, and 90.2% of completed interviews took place within six months of delivery.

During the interview, the 112-items Migrant Friendly Maternity Care Questionnaire (MFMCQ) was administered [19]. The MFMCQ covered a wide range of topics related to women’s maternity care experience, including their perceptions on communication with their maternal healthcare providers. It also included items on migration characteristics, such as the perceived proficiency in the Portuguese language.

### Context

In Portugal, international migrants (foreign-born individuals) made up 10.6% of the population by the end of 2019, while individuals with non-Portuguese nationality represented 5.7% [20]. Women with non-Portuguese nationality accounted for 12.7% of total births in Portugal with a much higher rate of live-births than women with Portuguese nationality (38 vs. 15 live-births per 1000 women, respectively)—showing a very positive contribution to the Portuguese demography [20].

The universal healthcare system in Portugal provides all women with free access to maternal health services, regardless of their country of birth or legal status [21]. Pregnant women typically seek public healthcare through their primary healthcare centers and family doctors unless their pregnancy is considered high-risk, in which case they will be referred to a specialist and be followed in a hospital.

Professional interpretation services are often not adequately accessible in Portuguese public healthcare [22]. Instead, ad-hoc interpreters (family, friends, untrained staff, etc.), machine translation services like Google Translate, or common second languages like English are frequently used to facilitate the communication between healthcare providers and non-Portuguese speaking patients.

### Study population

All baMBINO participants who received their entire pregnancy care in Portugal and conducted the full interview were considered eligible for the current study (*n* = 2712) (Fig. 1). The reason for excluding women with incomplete interviews is the large quantity of missing information, particularly that migration-related questions were posed at the end of the interview.

Women with complete interviews but no data on Portuguese proficiency level (*n* = 59) or perceived communication quality (*n* = 43) were excluded from the primary analysis but included after performing multiple imputation.

### Exposure measure

Our primary exposure was the self-reported proficiency in host-country language (Portuguese), categorized into native, full, intermediate, and limited [16].

Portuguese-born women were assumed to have native proficiency, while migrant women were asked to rate their proficiency in four components of the language: understanding, speaking, reading, and writing. The rating was a 4-point Likert scale: 0-no proficiency, 1-limited, 2-intermediate, and 3-full proficiency. We defined the overall proficiency score as the mode of each participant’s four components’ rating. Since only a few women had no proficiency (*n* = 25 in understanding, *n* = 39 in speaking, *n* = 43 in reading, and *n* = 57 in writing), we combined them into the limited proficiency category. When the set of four component ratings had a bimodal distribution (*n* = 77, e.g. 1-1-3-3), we assumed women to have intermediate proficiency. We also assumed Brazil-born women to have full proficiency since Portuguese is the primary spoken language in Brazil, but we could not assume the same for women born in Portuguese-speaking African countries (PALOP) due to the high number of other local languages in those countries. Since women’s oral proficiency (comprising only speaking and understanding) was consistent with their overall proficiency, we only focused on the overall proficiency as the main exposure in our study.

### Outcome measure

Our primary outcome was the perceived women-prenatal care provider communication quality (WPCQ). Women were asked to rate the accuracy of 9 items related to communication quality, using a 4-point Likert scale (0-always, 1-sometimes, 2-rarely, and 3-never), with three items focusing on healthcare professionals’ communication with them, and six items on their communication with the healthcare professionals:

- I understood the information provided by the healthcare professionals.
- I felt comfortable asking about things I did not understand.
- I felt my worries were taken seriously by the healthcare professionals.
- The healthcare professionals asked me if I had any questions.
- The healthcare professionals spent enough time providing explanations.
- The healthcare professionals kept me informed about what was happening.
- The healthcare professionals were very encouraging and reassuring.
- The healthcare professionals were rushed.
- The healthcare professionals made decisions without my wishes being taken into account.

We then performed a principal component analysis to evaluate the dimensionality of these nine items. Based on the number of components with eigenvalues > 1.0 and the respective scree plot, we concluded that a one-component solution was the most appropriate, explaining 34.9% of the total variance. We retained all items as all factor loadings were higher than 0.3 (ranging from 0.35 to 0.76), and the reliability coefficient did not improve when any of the items were dropped. The Cronbach’s alpha coefficient of the items set was 0.73, indicating satisfactory internal reliability. The WPCQ score was then calculated by adding up the scores of all items.

To maintain consistency in the directionality of responses, we reversed the coding of the response scale for the last two items (i.e., 0-never to 3-always). The final score ranged from 0 to 27, with zero indicating “optimal” perceived communication quality and higher scores indicating “suboptimal” perceptions of communication quality.

### Covariates

Covariates consisted of migration-related, sociodemographic, and obstetric characteristics. Migration-related factors included: maternal region of birth (Europe [including Portugal], Africa, Americas, Asia), and length of stay in Portugal (≤ 5, 5–10, > 10 years). Sociodemographic factors included: maternal age (18–24, 25–34, ≥ 35 years), marital status (partner [married or civil union], no partner [single, divorced, or widowed]), highest educational level achieved (post-secondary, upper-secondary, less than upper-secondary [lower secondary, primary, or no education]), monthly income per person in the household (< 500, 500–1000, > 1000 Euros), healthcare system used (only national health services, extra individual health insurance plan), and administrative health region (Lisbon, Center, North, Algarve).

Obstetric characteristics included: parity (primiparous, multiparous), smoking during the index pregnancy (yes, no), any complications during the index pregnancy (yes, no), and adequacy of prenatal care utilization (adequate, intermediate/inadequate [Initiation of prenatal care after 12 gestational weeks or less than 80% of the recommended number of visits according to gestational age]) [23].

### Statistical analysis

We reported the frequencies and proportions of participants’ characteristics and compared them across language proficiency groups using the Chi-square or Fisher exact tests. We also reported the median WPCQ scores (along with the 25^th^ and 75^th^ percentiles) across participants’ characteristics and compared their rank sum using the Mann–Whitney and Kruskal–Wallis tests.

To estimate the associations of language proficiency and other explanatory variables with WPCQ, we compared the fit of four models: Poisson, negative binomial, zero-inflated Poisson, and zero-inflated negative binomial (ZINB). The ZINB model had a superior relative fit based on its estimates of precision (difference between the predicted and observed probabilities of each count outcome for each distribution), Akaike information criterion (AIC), and Bayesian information criterion (BIC) (Fig. S1). ZINB had the best fit because it accounted for the inflation of zeros/”optimal” WPCQ scores (i.e., the excess of zeros compared to what standard Poisson or negative binomial distributions can predict) and the overdispersion in the distribution of WPCQ.

The ZINB model assumes that the excess zeros are generated by a separate process from the count values and can be modelled independently. Therefore, ZINB generated and combined two components [24]: 1- a zero-inflation component (logit model for predicting the probability of excess zeros vs. all other scores), and 2- a count component (negative-binomial regression model for predicting WPCQ scores giving less weight/controlling for the probability of excess zeros). The excess zeros would refer to a group of women who will presumably always report optimal communication scores in all items considered. Results were reported as crude and adjusted odds ratios (OR) for the logit component, and incidence rate ratios (IRR) for the negative binomial component, along with the respective 95% confidence intervals (CI).

We chose the covariates included in the final multivariable model a priori based on their relevance according to the literature and the best statistical fit for the model. Covariates included maternal age, highest educational degree attained, healthcare system used, administrative health region, parity, and complications during the index pregnancy.

Among the variables included in the models, the percentage of missing data ranged from 0 to 7.9%. Missing values were handled using multiple imputations by chained equations with binary, multinomial, and ordered logistic regression imputation models for binary, nominal, and ordinal variables, respectively. All the variables considered in the analytic model were included in the imputation model, in addition to prenatal care utilization adequacy, monthly income, marital status, and smoking during pregnancy as auxiliary variables. The outcome variable was imputed as nine individual items and then combined into a score. Associations were estimated within each of the 50 imputed data sets generated with 20 iterations and results were pooled in a single estimate, according to Rubin’s rules.

We compared the characteristics of included women with and without a complete interview. We used the inverse probability weighting approach to assess the impact of loss to follow-up on our results [25]. We estimated the probability of being included in the final sample for eligible women as a function of the region of birth, age, marital status, highest education level attained, administrative health region, parity, and smoking during pregnancy. We then assigned each participant in the analysis with a weight corresponding to the inverse of the calculated probability. All analyses were repeated using the calculated weights.

We finally performed two sensitivity analyses: 1) without assuming full proficiency for Brazilian women, and 2) additionally adjusting for women’s length of stay in Portugal in order to disentangle the effect of language proficiency from that of cultural influences or other acculturation factors.

All data were analyzed using Stata 17.0 (StataCorp LP, College Station, TX, USA). Statistical significance was set at *p* < 0.05.

## Results

Among 2712 eligible women, 2610 (96.2%) had complete information on language proficiency and communication quality perceptions and were included in the main analysis (Fig. 1). Significant differences were observed in most maternal characteristics according to eligible women’s inclusion status in the final sample. Eligible women not included in the sample were more often African migrants, younger, single, of low education, living in Lisbon, multiparous, smokers during pregnancy, and inadequate users of prenatal care (Table S1).

Migrant women made up 46.4% of the complete-case analysis; out of those, 732 (60.5%) had full proficiency, 338 (27.9%) had intermediate proficiency, and 140 (11.6%) had limited proficiency in Portuguese.

Table 1 compares the migration-related, sociodemographic, and obstetric characteristics of participants across language proficiency levels. All maternal characteristics varied significantly across language proficiency levels except for parity and complications during pregnancy. Compared to other proficiency levels, women with limited skills stood out for being more often: born in Asia, living in Portugal for ≤ 5 years, younger (18–34 years old), with a partner, of low monthly income, sole users of the national health services, and inadequate users of prenatal care.

**Table 1:** Maternal characteristics by host-country language proficiency and perceived communication quality scores (*n* = 2610)

Characteristics	Language Proficiency				*P*	Communication quality score	P**
	**Native** **(*n* = 1400)**	**Full** **(*n* = 732)**	**Intermediate** **(*n* = 338)**	**Limited** **(*n* = 140)**		**Median (P25,P75)**	
	**n (%)**	**n (%)**	**n (%)**	**n (%)**			
**Migration-related characteristics**							
**Language Proficiency (*n* = 2610)**	-	-	-	-			
Native	-	-	-	-		2 (0,4)	
Full proficiency	-	-	-	-	-	2 (0,5)	** < .001**
Intermediate proficiency	-	-	-	-		3 (0,5)	
Limited proficiency	-	-	-	-		4 (2,7)	
**Region of birth (*n* = 2610)**							
Europe (including Portugal)	1400 (0.0)	146 (20.0)	94 (27.8)	43 (30.7)		2 (0,4)	
Africa	-	274 (37.4)	218 (64.5)	52 (37.2)	< .001*	3 (0,5)	** < .001**
Americas^a^	-	310 (42.3)	14 (4.1)	1 (0.7)		2 (0,5)	
Asia	-	2 (0.3)	12 (3.6)	44 (31.4)		3 (2,7)	
**Length of stay in Portugal (*n* = 1204)**							
≤ 5 years	-	150 (20.6)	114 (34.0)	100 (71.5)		3 (1,6)	
5–10 years	-	182 (25.0)	127 (37.9)	31 (22.1)	** < .001**	3 (0,6)	** < .001**
> 10 years	-	397 (54.4)	94 (28.1)	9 (6.4)		2 (0,4)	
**Sociodemographic characteristics**							
**Age (years) (*n* = 2610)**							
18–24	180 (12.9)	121 (16.5)	59 (17.5)	29 (20.7)		2 (0,5)	
25–34	775 (55.3)	387 (52.9)	193 (57.1)	87 (62.2)	**.001**	2 (0,4)	**.032**
≥ 35	445 (31.8)	224 (30.6)	86 (25.4)	24 (17.1)		2 (0,4)	
**Marital status (*n* = 2610)**							
Partner	1062 (75.9)	535 (73.1)	221 (65.4)	126 (90.0)	** < .001**	2 (0,4)	.292
No Partner	338 (24.1)	197 (26.9)	117 (34.6)	14 (10.0)		2 (0,4)	
**Highest education level attained (*n* = 2609)**							
Post-secondary (> 12^th^ grade)	555 (39.6)	212 (29.0)	89 (26.3)	50 (36.0)		2 (1,4)	
Upper-secondary (12^th^ grade)	482 (34.4)	355 (48.5)	102 (30.2)	31 (22.3)	** < .001**	2 (0,5)	.117
< Upper-secondary	363 (26.0)	165 (22.5)	147 (43.5)	58 (41.7)		2 (0,4)	
**Monthly household income (*n* = 2497)**							
< 500 €/person	579 (43.0)	454 (64.8)	247 (78.2)	108 (80.6)		2 (0,5)	
500–1000 €/person	645 (47.9)	224 (31.9)	61 (19.3)	24 (17.9)	** < .001**	2 (0,4)	.172
> 1000 €/person	122 (9.1)	23 (3.3)	8 (2.5)	2 (1.5)		2 (1,4)	
**Healthcare system used (*n* = 2554)**							
National health services	743 (54.3)	528 (73.2)	279 (84.0)	118 (88.7)	** < .001**	2 (0,5)	.290
Extra individual health insurance plan	625 (45.7)	193 (26.8)	53 (16.0)	15 (11.3)		2 (0,4)	
**Administrative health region (*n* = 2610)**							
Lisbon	769 (54.9)	438 (59.9)	251 (74.3)	90 (64.3)		2 (0,5)	
Center	185 (13.2)	80 (10.9)	16 (4.7)	17 (12.1)	** < .001**	2 (0,4)	** < .001**
North	357 (25.5)	161 (22.0)	41 (12.1)	21 (15.0)		2 (0,3)	
Algarve	89 (6.4)	53 (7.2)	30 (8.9)	12 (8.6)		3 (0,5)	
**Obstetric characteristics**							
**Parity (*n* = 2500)**							
Primiparous	685 (50.8)	336 (47.9)	135 (43.0)	65 (47.4)	.077	2 (0,5)	** < .001**
Multiparous	662 (49.2)	366 (52.1)	179 (57.0)	72 (52.6)		2 (0,4)	
**Smoking during pregnancy (*n* = 2555)**							
No	1164 (84.9)	668 (92.6)	314 (95.7)	128 (94.8)	** < .001**	2 (0,4)	**.**087
Yes	207 (15.1)	53 (7.4)	14 (4.3)	7 (5.2)		2 (0,4)	
**Complications during pregnancy**^b^** (*n* = 2553)**							
No	978 (71.4)	502 (70.0)	234 (71.1)	97 (70.3)	.919	2 (0,4)	**.014**
Yes	391 (28.6)	215 (30.0)	95 (28.9)	41 (29.7)		2 (0,5)	
**Adequacy of prenatal care utilization (*n* = 2405)**							
Adequate	1064 (82.2)	507 (76.4)	214 (68.8)	83 (61.5)	** < .001**	2 (0,4)	** < .001**
Intermediate/Inadequate^c^	231 (17.8)	157 (23.6)	97 (31.2)	52 (38.5)		3 (0,5)	

*P25* 25^th^ percentile, *P75* 75^th^ percentile

^*^Native women were not included in this comparison

^**^Kruskal–Wallis or Mann–Whitney test

^a^Oceania was included in the Americas category due to small numbers

^b^Complications during pregnancy were retrieved from clinical records and included: high blood pressure,preeclampsia,gestational diabetes,acute pyelonephritis,placenta praevia,placental abruption,and other rare complications

^c^Intermediate/Inadequate utilization of prenatal care is based on the Adequacy of Prenatal Care Utilization (APNCU) Index and it refers to initiation of prenatal care after 12 gestational weeks or having less than 80% of the recommended number of prenatal visits according to gestational age

Overall, 757 women (29%) perceived the communication quality with their prenatal healthcare providers as “optimal” (i.e., WPCQ = 0). The median perceived communication score for the sample was 2 ([25^th^,75^th^ percentile] 0,4). Women with native, full, intermediate, and limited language proficiencies had a median communication score of 2 (0,4), 2 (0,5), 3 (0,5), and 4 (2,7), respectively (Table 1). We observed differences in WPCQ scores by women’s maternal region of birth, length of stay in Portugal, age, region, parity, complications during pregnancy, and adequacy of prenatal care utilization.

We presented the results of the ZINB regression analysis for WPCQ perceptions in Table 2. The ZINB’s zero-inflation component showed that women’s language proficiency did not influence the probability of always rating communication quality with prenatal care providers as “optimal” (best communication score [zero] in all nine items considered). This result was robust even after adjusting for age, education, type of healthcare system used, region, parity, and complications during pregnancy. However, as shown in ZINB’s count component, language proficiency was a significant predictor of “suboptimal/non-zero” WPCQ scores. Women with full (aIRR 1.35 [95% CI 1.22,1.50]), intermediate (aIRR 1.41 [95% CI 1.23,1.61]), and limited (aIRR 1.72 [95% CI 1.45,2.05]) language proficiencies were increasingly more likely to have lower perceived communication quality when compared to natives (Table 2). Analyses after multiple imputation and inverse probability weights showed similar results to those of complete-case and unweighted analyses, respectively (Table S2). Similar results were also obtained when we performed the analysis using Brazilian women’s reported language proficiency without assumptions and when we additionally adjusted for the length of stay in Portugal (Table S3, S4).

**Table 2:** Zero-inflated negative binomial regression models estimating the association between language proficiency and perceived communication quality scores (n = 2407)

	**Zero-inflated part**								**Negative binomial part**							
	**Before multiple imputation**				**After multiple imputation**				**Before multiple imputation**				**After multiple imputation**			
	**OR**	**95% CI**	**aOR**	**95% CI**	**OR**	**95% CI**	**aOR**	**95% CI**	**IRR**	**95% CI**	**aIRR**	**95% CI**	**IRR**	**95% CI**	**aIRR**	**95% CI**
**Language proficiency (*n* = 2610)**																
Native (ref)	1.00	—	1.00	—	1.00	—	1.00	—	1.00	—	1.00	—	1.00	—	1.00	—
Full	1.35	(0.93,1.95)	1.16	(0.79,1.71)	1.37	(0.95,1.98)	1.24	(0.86,1.79)	**1.36**	**(1.22,1.51)**	**1.34**	**(1.20,1.50)**	**1.37**	**(1.24,1.52)**	**1.35**	**(1.22,1.50)**
Intermediate	1.51	(0.98,2.34)	1.41	(0.85,2.33)	**1.62**	**(1.05,2.50)**	1.47	(0.94,2.30)	**1.46**	**(1.29,1.65)**	**1.37**	**(1.18,1.59)**	**1.48**	**(1.30,1.69)**	**1.41**	**(1.23,1.61)**
Limited	0.49	(0.18,1.32)	0.51	(0.21,1.27)	0.55	(0.22,1.39)	0.58	(0.26,1.33)	**1.71**	**(1.49,1.97)**	**1.71**	**(1.45,2.00)**	**1.78**	**(1.51,2.11)**	**1.72**	**(1.45,2.05)**
**Age (*n* = 2610)**																
18–24	0.76	(0.43,1.33)	0.86	(0.54,1.38)	0.78	(0.44,1.36)	0.81	(0.51,1.29)	1.03	(0.91,1.15)	1.02	(0.90,1.16)	1.04	(0.92,1.18)	1.01	(0.89,1.14)
25–34 (ref)	1.00	—	1.00	—	1.00	—	1.00	—	1.00	—	1.00	—	1.00	—	1.00	—
≥ 35	1.23	(0.85,1.77)	1.15	(0.80,1.65)	1.23	(0.85,1.78)	1.18	(0.83,1.66)	0.96	(0.86,1.06)	1.02	(0.92,1.14)	0.94	(0.85,1.04)	0.98	(0.89,1.08)
**Highest education level attained (*n* = 2609)**																
Post-secondary (> 12^th^ grade) (ref)	1.00	—	1.00	—	1.00	—	1.00	—	1.00	—	1.00	—	1.00	—	1.00	—
Upper-secondary (12^th^ grade)	**2.38**	**(1.30,4.33)**	**2.50**	**(1.31,4.77)**	**2.50**	**(1.34,4.67)**	**2.63**	**(1.48,4.66)**	1.07	(0.96,1.19)	1.06	(0.94,1.20)	1.06	(0.95,1.17)	1.02	(0.91,1.13)
< Upper-secondary	**3.61**	**(1.96,6.63)**	**3.53**	**(1.91,6.54)**	**3.84**	**(2.04,7.25)**	**3.74**	**(2.11,6.63)**	**1.17**	**(1.04,1.30)**	1.11	(0.97,1.26)	**1.19**	**(1.06,1.33)**	1.11	(0.99,1.25)
**Healthcare system used (*n* = 2554)**																
National health services	1.00	—	1.00	—	1.00	—	1.00	—	1.00	—	1.00	—	1.00	—	1.00	—
Extra individual health insurance plan	0.64	(0.42,0.97)	0.89	(0.58,1.38)	**0.61**	**(0.39,0.94)**	0.97	(0.66,1.43)	**0.82**	**(0.74,0.90)**	0.94	(0.84,1.05)	**0.83**	**(0.75,0.91)**	0.96	(0.86,1.06)
**Administrative health region (*n* = 2610)**																
Lisbon and Tagus valley (ref)	1.00	—	1.00	—	1.00	—	1.00	—	1.00	—	1.00	—	1.00	—	1.00	—
Center	1.30	(0.76,2.21)	1.58	(0.93,2.68)	1.10	(0.64,1.91)	1.55	(0.95,2.55)	**0.80**	**(0.69,0.93)**	**0.85**	**(0.73,0.99)**	**0.82**	**(0.71,0.94)**	0.87	(0.76,1.00)
North	1.37	(0.91,2.05)	1.37	(0.90,2.08)	1.28	(0.86,1.91)	1.45	(0.98,2.16)	**0.84**	**(0.74,0.95)**	**0.84**	**(0.74,0.94)**	**0.83**	**(0.75,0.93)**	**0.86**	**(0.77,0.95)**
Algarve	0.99	(0.50,1.95)	0.98	(0.51,1.88)	0.99	(0.50,1.96)	1.07	(0.58,1.95)	1.01	(0.86,1.18)	1.06	(0.90,1.24)	0.99	(0.84,1.18)	1.00	(0.85,1.18)
**Parity (*n* = 2500)**																
Multiparous (ref)	1.00	—	1.00	—	1.00	—	1.00	—	1.00	—	1.00	—	1.00	—	1.00	—
Primiparous	**0.53**	**(0.36,0.77)**	**0.68**	**(0.47,0.99)**	**0.52**	**(0.35,0.76)**	0.71	(0.50,1.02)	1.04	(0.94,1.14)	1.06	(0.96,1.18)	1.02	(0.93,1.12)	1.07	(0.98,1.18)
**Complications during pregnancy**^a^ **(*n* = 2553)**																
No (ref)	1.00	—	1.00	—	1.00	—	1.00	—	1.00	—	1.00	—	1.00	—	1.00	—
Yes	0.71	(0.47,1.07)	0.73	(0.50,1.07)	0.69	(0.45,1.06)	**0.68**	**(0.47,0.97)**	1.04	(0.94,1.14)	1.06	(0.96,1.17)	1.03	(0.94,1.14)	1.05	(0.95,1.15)

*n* = 2407 in the adjusted models before multiple imputation, and *n* = 2712 in the adjusted models after multiple imputation

*OR* crude odds ratios, *aOR* adjusted odds ratios, *IRR* crude incidence rate ratios, *aIRR* adjusted incidence rate ratios

^a^Complications during pregnancy were retrieved from clinical records and included: high blood pressure,preeclampsia,gestational diabetes,acute pyelonephritis,placenta praevia,placental abruption,and other rare complications

## Discussion

Our findings showed that native and migrant women had overall positive perceptions of prenatal communication quality in Portugal, with 29% of women rating all WPCQ aspects as “optimal.” Women’s host-country language proficiency did not influence the probability of always perceiving communication quality with their prenatal care providers as “optimal”. In other words, most women who perceived WPCQ as “optimal” did so regardless of their language skills. Among the rest of the women, however, language proficiency was associated with WPCQ rating independently of sociodemographic and obstetric factors; as migrant women’s proficiency decreased, so did their perception of the provided communication quality compared to natives.

Our results generally corroborate the findings of qualitative studies conducted in the United States, Canada, the United Kingdom, Switzerland, Sweden, Norway, Finland, and Portugal, which asserted the importance of migrants’ language proficiency or language concordance with healthcare professionals in promoting positive perceptions of communication quality in maternity care [26–35]. In those studies, language barriers were shown to hinder various aspects of communication quality in perinatal care, such as understanding of information and procedures, particularly when involving medical terms; feeling informed; asking questions; expressing feelings, concerns, symptoms, needs, and preferences; and being involved in decision-making [26–35].

On the other hand, quantitative data on the association between language proficiency and perceived communication quality in the perinatal care context was extremely scarce. To our knowledge, only one recent study investigated host-country language proficiency as one of the factors associated with the perceived understanding of the information provided by maternity staff among newly arrived migrants in Norway [36]. In line with our findings, Bains et al. noted that limited host-country language proficiency was associated with poorer perceived understanding of information (aOR 2.14, 95% CI (1.14,4.02)) when compared to high proficiency [36]. It is worth noting that Bains et al. also employed the MFMCQ in their research [36].

In contrast to earlier findings on the topic, implying a somehow linear relationship between language skills and WPCQ, the present study highlighted an important distinction: some women always have an “optimal” WPCQ score regardless of their language proficiency, whereas, for the rest of women, decreasing language proficiency is a significant predictor of “suboptimal” communication quality perceptions. A possible explanation for those findings might be that, for women to be completely satisfied with all aspects of WPCQ, factors like education or parity could be more important than language proficiency—which only comes into play when women are already incompletely satisfied. Indeed, some previous studies indicated a link between educational attainment and quality ratings of maternity services, with women of high educational level having higher expectations and, thus, perceiving a lower quality of care [37–39]. Another study on women’s perceptions of prenatal care quality reported a limited impact of sociodemographic factors, but a significant positive association with parity—multiparous women had their expectations trimmed by previous contacts with maternity care [40]. A note of caution is due here since there is a paucity of consistent evidence on the determinants of perceived WPCQ. Another possible explanation for the distinction reported in our findings could have to do with response bias and personality traits. Response bias is a systematic deviation of responses from actual values that is unrelated to the construct of interest but rather to personality traits and is especially prevalent in self-report Likert-scale measurements [41]. We speculate that women with agreeable personality traits—characterized by a tendency to be cooperative, considerate, and empathetic—may potentially be more likely to always perceive communication quality as “optimal” if they feel that they received caring and compassionate care from prenatal care providers, regardless of their language skills. It could also be that “optimal” perceptions could have, at least partly, been an indicator of courtesy or social desirability biases which occur when individuals tend to conceal unhappiness with a service to be courteous to the interviewer and respond in a way that they perceive is more desirable [41, 42]. This type of bias is particularly relevant when interviewers are believed to be affiliated with the service evaluated [42]. Our interviewers were public health researchers and could have been mistakenly associated with public maternity health services by some of our respondents. If these speculations are correct, it would imply that 1) language proficiency is not significantly associated with belonging to the group of women who have agreeable personality traits and/or are prone to response bias—as other factors like educational attainment may be more important—and 2) language proficiency is an important predictor of perceived communication quality for women who do not possess these traits. However, since we do not have any data on personality characteristics, we cannot verify these hypotheses.

### Practice implications and future research

The present study has been one of the first attempts to quantitatively examine the effect of host-country language command, a potentially modifiable factor, on women’s perceptions of women-prenatal care provider communication quality—an essential aspect of person-centered quality prenatal care.

As the global population of international migrants and ethnic minorities—including a large proportion of women of reproductive age—grows [43], so does the potential role of language in prenatal healthcare encounters. Our results suggest that in order to improve prenatal care quality and address disparities in women-provider communication experiences in prenatal care, efforts should account not only for sociodemographic and health characteristics of pregnant women but also for their language skills—paying particular attention to those with no or limited proficiency. To achieve that, it would be vital to firstly increase prenatal care providers’ awareness of the linguistic and cultural needs of migrant women and support them with the training and skills necessary to address those individualized needs and to provide a culturally safe environment [44]. This is especially pertinent when considering that maternity units directors tend to rate perinatal healthcare quality higher than migrant women, as we recently reported in a recent Portuguese study [45]. A range of measures could also facilitate communication barriers, including, but not limited to, increasing access to professional interpretation services, providing translated written or audio material, dedicating sufficient time for consultations, and optimizing the use of digital communication support within perinatal care [15, 32].

Our findings are particularly relevant given that previous studies on the role of language proficiency in perinatal healthcare and outcomes have also revealed a link between limited language skills and several adverse outcomes, including inadequate prenatal care utilization, obstetric trauma, and postpartum depressive symptoms [16, 23, 46]. Taken together, our findings, along with those of previous studies, would lay the groundwork for future research into the pathways linking language proficiency to perinatal health outcomes and pave the way for potential intervention studies. This is particularly pertinent in a country with one of Europe's lowest fertility rates, where births among migrant women are increasing and partially offsetting the fertility decline incurred by population aging [20].

Finally, to better understand different facets of the complex notion of perceived WPCQ, further research could usefully examine non-verbal aspects of communication in prenatal care encounters of migrant women, such as body language, as well as explore the perspectives of prenatal care providers on their encounters with migrant women with language barriers.

### Strengths and limitations

One of the main strengths of our study is that a large sample of women was interviewed in their language of choice, thanks to a multi-lingual team of interviewers and professional interpretation services – this enabled us to include migrant women with poor language competencies who are frequently excluded in migrant health literature. We also used a more comprehensive approach in our measurement of the host-country language proficiency than most studies by combining different language components (understanding, speaking, reading, writing) in a scale of self-rated proficiency levels (native, full, intermediate, limited), rather than simply categorizing women to speakers or non-speakers based on their country of origin, nationality, or preferred language of interview. We used a subjective measure of language proficiency since we believe that perceived language barriers could be more relevant than objective ones in the context of perceived communication quality. Additionally, from a public health perspective, our results would implicate collecting migrant women’s perceived language proficiency in routine practice, which would be much more feasible than objectively testing their skills. Finally, we used advanced statistical methods to respond to the complexity of the investigated topic. We fitted a zero‐inflated negative binomial (ZINB) model to allow for the excess number of women with an “optimal” perception of prenatal care communication quality, which could be considered a unique approach to addressing our research question.

Some potential limitations of this study should be considered. Characteristics of women included in the analysis differed significantly from those of women at baseline due to a relatively high loss to follow-up rate. However, our results were consistent after adjusting for the inverse participation probability weights, which attest to the robustness of our conclusions. Despite professional interpretation services not being adequately functional in the healthcare settings in Portugal [22], prenatal healthcare professionals could have made efforts to reduce the language barriers experienced by women using a second language or machine translating services. We had no comprehensive information regarding those potential efforts. Subsequently, the effect of language proficiency on communication quality perception might have been attenuated in our research, as such facilitators were not accounted for. On a related note, we also did not have information about women’s health literacy or competency in English – a frequently common second language between migrants and health providers. However, we considered that education level could act as a good proxy for both.

## Conclusions

Perceptions of women-prenatal care provider communication quality worsen with women’s decreasing host-country language proficiency. The findings of this study shed new light on health communication quality determinants among migrant women and call for greater efforts to ensure the provision of linguistically competent prenatal care for women with limited language proficiency, especially given their previously established higher likelihood to have inadequate utilization of prenatal care, and adverse perinatal health outcomes.

## Supplementary Information

**Additional file 1: Figure S1.** Comparison of observed versus predicted probabilities, and statistical fit indicators, of Poisson (PRM), Negative Binomial (NBRM), Zero-inflated Poisson (ZIP), and Zero-inflated Negative Binomial (ZINB) regression adjusted models. **Table S1.** Maternal characteristics by participation status among eligible women who consented to participate (*n*=4978). **Table S2.** Zero-inflated negative binomial regression models estimating the association between language proficiency and perceived communication quality scores after inverse probability weighting (*n*=2367). **Table S3.** Zero-inflated negative binomial regression models estimating the association between language proficiency and perceived communication quality scores with and without assuming full proficiency of Brazilian women. **Table S4.** Zero-inflated negative binomial regression models estimating the association between language proficiency and perceived communication quality scores before and after additionally adjusting for length of stay in Portugal.

## Data Availability

The data that supports the findings of this study is available upon request from the Institute of Public Health of the University of Porto (ISPUP).

## Abbreviations

WPCQ: Women-prenatal care provider communication quality.
aIRR: Adjusted incidence rate ratios.
aOR: Adjusted odds ratio.
CI: Confidence intervals.
MFMCQ: Migrant Friendly Maternity Care Questionnaire.
PALOP: Portuguese-speaking African countries.
ZINB: Zero-inflated negative binomial
