# Supplementary material for: Migrant and native women’s perceptions of prenatal care communication quality: the role of host-country language proficiency
Source: BMC Public Health. 2023 Feb 9;23:295. doi: 10.1186/s12889-023-15154-4 (PMC9909846; doi:10.1186/s12889-023-15154-4)
Supplement: Supplementary file 1 — Additional file 1: Figure S1. Comparison of observed versus predicted probabilities, and statistical fit indicators, of Poisson (PRM), Negative Binomial (NBRM), Zero-inflated Poisson (ZIP), and Zero-inflated Negative Binomial (ZINB) regression adjusted models. Table S1. Maternal characteristics by participation status among eligible women who consented to participate (n=4978). Table S2. Zero-inflated negative binomial regression models estimating the association between language proficiency and perceived communication quality scores after inverse probability weighting (n=2367). Table S3. Zero-inflated negative binomial regression models estimating the association between language proficiency and perceived communication quality scores with and without assuming full proficiency of Brazilian women. Table S4. Zero-inflated negative binomial regression models estimating the association between language proficiency and perceived communication quality scores before and after additionally adjusting for length of stay in Portugal. [file 12889_2023_15154_MOESM1_ESM.docx]

**Figure S1.** Comparison of observed versus predicted probabilities, and statistical fit indicators, of Poisson (PRM), Negative Binomial (NBRM), Zero-inflated Poisson (ZIP), and Zero-inflated Negative Binomial (ZINB) regression adjusted models


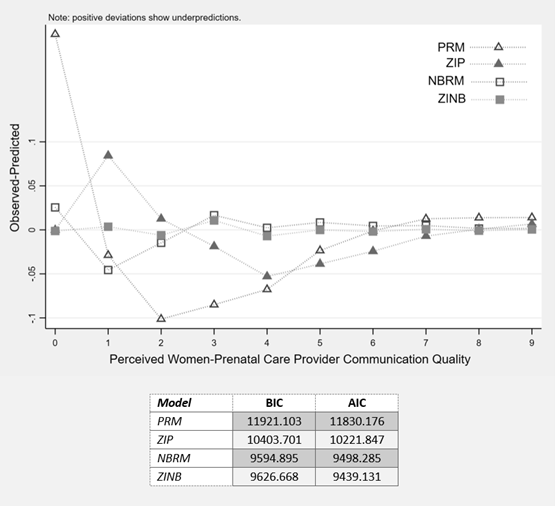


| **Table S1.** Maternal characteristics by participation status among eligible women who consented to participate (n=4978) | | | |
| --- | --- | --- | --- |
| **Characteristics** | **Eligible non-participantsᵅ**  **(*n* = 2368)** | **Participants**  **(*n* = 2610)** | ***p*** |
|  | ***n* (%)** | ***n* (%)** |  |
| **Sociodemographic characteristics** |  |  |  |
| **Migrant** (*n* = 4978) | 1291 (54.5) | 1210 (46.4) | **<0.001** |
| **Region** **of birth** (*n* = 4978) |  |  |  |
| Europe (including Portugal) | 1289 (54.4) | 1683 (64.6) |  |
| Africa | 789 (33.3) | 544 (20.8) | **<0.001** |
| Americaᵇ | 222 (9.4) | 325 (12.4) |  |
| Asia | 68 (2.9) | 58 (2.2) |  |
| **Age** **(years)** (*n* = 4955) |  |  |  |
| 18-24 | 455 (19.4) | 389 (14.9) | **<0.001** |
| 25-34 | 1306 (55.7) | 1442 (55.2) |  |
| ≥35 | 584 (24.9) | 779 (29.9) |  |
| **Marital status (no partner)** (*n* = 4944) | 703 (30.1) | 666 (25.5) | **<0.001** |
| **Highest education level attained** (*n* = 4765) |  |  |  |
| Post-secondary (>12^th^ grade) | 574 (26.7) | 906 (34.7) | **<0.001** |
| Upper secondary (12^th^ grade) | 755 (35.0) | 970 (37.2) |  |
| < Upper-secondary | 827 (38.3) | 733 (28.1) |  |
| **Administrative health region** (*n* = 4978) |  |  |  |
| Lisbon | 1703 (71.9) | 1548 (59.3) | **<0.001** |
| Center | 168 (7.1) | 298 (11.4) |  |
| North | 400 (16.9) | 580 (22.2) |  |
| Algarve | 97 (4.1) | 184 (7.1) |  |
| **Obstetric characteristics** |  |  |  |
| **Primiparous** (*n* = 4730) | 969 (43.5) | 1221 (48.8) | **<0.001** |
| **Smoking during pregnancy** (*n* = 4856) | 330 (14.3) | 281 (11.0) | **<0.001** |
| **Complications during pregnancy ᶜ** (*n* = 4863) | 642 (27.8) | 742 (29.1) | 0.33 |
| **Twin pregnancy** (*n* = 4978) | 51 (2.2) | 43 (1.7) | 0.19 |
| **Intermediate/Inadequate prenatal care utilization ᵈ (***n* = 4511**)** | 789 (37.5) | 537 (22.3) | **<0.001** |
| ^a^ Eligible non-participants included women who were: unreachable, did not complete the interview, or had missing information on language proficiency or communication quality, and excluded refusals. ᵇ Oceania was included in the Americas category due to small numbers. ᶜ Complications during pregnancy were retrieved from clinical records and included: high blood pressure, preeclampsia, gestational diabetes, acute pyelonephritis, placenta praevia, placental abruption, and other rare complications. ᵈ Intermediate/Inadequate utilization of prenatal care is based on the Adequacy of Prenatal Care Utilization (APNCU) Index and it refers to initiation of prenatal care after 12 gestational weeks or having less than 80% of the recommended number of prenatal visits according to gestational age. | | | |

| **Table S2.** Zero-inflated negative binomial regression models estimating the association between language proficiency and perceived communication quality scores after inverse probability weighting (n=2367). | | | | | | | | | |
| --- | --- | --- | --- | --- | --- | --- | --- | --- | --- |
|  | **Communication quality score** | **Zero-inflated part** | | | | **Negative binomial part** | | | |
|  | **Weighted Mean (95% CI)** | **OR** | **95% CI** | **aOR** | **95% CI** | **IRR** | **95% CI** | **aIRR** | **95% CI** |
| **Language proficiency** |  |  |  |  |  |  |  |  |  |
| Native (ref) | 2.5 (2.31,2.63) | 1.00 | — | 1.00 | — | 1.00 | — | 1.00 | — |
| Full | 3.1 (2.85,3.40) | 1.24 | (0.86,1.80) | 1.08 | (0.73,1.61) | **1.32** | **(1.18,1.47)** | **1.28** | **(1.14,1.43)** |
| Intermediate | 3.2 (2.84,3.63) | 1.48 | (0.95,2.31) | 1.31 | (0.79,2.17) | **1.41** | **(1.24,1.61)** | **1.33** | **(1.15,1.54)** |
| Limited | 4.5 (3.88,5.13) | 0.48 | (0.18,1.32) | 0.54 | (0.22,1.33) | **1.67** | **(1.44,1.93)** | **1.62** | **(1.37,1.90)** |
| **OR**: weighted crude odds ratios; **aOR**: weighted adjusted odds ratios ; **IRR**: weighted crude incidence rate ratios; **aIRR**: weighted adjusted incidence rate ratios.  Models were based on complete-case analysis. | | | | | | | | | |

| **Table S3.** Zero-inflated negative binomial regression models estimating the association between language proficiency and perceived communication quality scores with and without assuming full proficiency of Brazilian women. | | | | | | | | |
| --- | --- | --- | --- | --- | --- | --- | --- | --- |
|  | **Zero-inflated part** | | | | **Negative binomial part** | | | |
|  | **Without assuming Brazilian women fluency** | | **Assuming Brazilian women fluency** | | **Without assuming Brazilian women fluency** | | **Assuming Brazilian women fluency** | |
|  | **aOR^a^** | **95% CI** | **aOR^b^** | **95% CI** | **aIRR^a^** | **95% CI** | **aIRR^b^** | **95% CI** |
| **Language proficiency (n=2610)** |  |  |  |  |  |  |  |  |
| Native (ref) | 1.00 | — | 1.00 | — | 1.00 | — | 1.00 | — |
| Full | 1.16 | (0.78,1.70) | 1.16 | (0.79,1.71) | **1.33** | **(1.19,1.50)** | **1.34** | **(1.20,1.50)** |
| Intermediate | 1.42 | (0.90,2.22) | 1.41 | (0.85,2.33) | **1.36** | **(1.19,1.57)** | **1.37** | **(1.18,1.59)** |
| Limited | 0.50 | (0.21,1.21) | 0.51 | (0.21,1.27) | **1.75** | **(1.49,2.05)** | **1.71** | **(1.45,2.00)** |
| **aOR**: adjusted odds ratios; **aIRR**: adjusted incidence rate ratios.  **^a^** Original models adjusted for age, highest educational level attained, healthcare system used, administrative health region, parity, and complications during pregnancy, assuming all Brazilian women to have full proficiency, n=2407**.**  **^b^** Models adjusted for age, highest educational level attained, healthcare system used, administrative health region, parity, and complications during pregnancy, without assuming all Brazilian women to have full proficiency, n=2367.  Models were based on complete-case analysis. | | | | | | | | |

| **Table S4.** Zero-inflated negative binomial regression models estimating the association between language proficiency and perceived communication quality scores before and after additionally adjusting for length of stay in Portugal. | | | | | | | | |
| --- | --- | --- | --- | --- | --- | --- | --- | --- |
|  | **Zero-inflated part** | | | | **Negative binomial part** | | | |
|  | **Before adjusting for length of stay** | | **After adjusting for length of stay** | | **Before adjusting for length of stay** | | **After adjusting for length of stay** | |
|  | **aOR^a^** | **95% CI** | **aOR^b^** | **95% CI** | **aIRR^a^** | **95% CI** | **aIRR^b^** | **95% CI** |
| **Language proficiency** |  |  |  |  |  |  |  |  |
| Native (ref) | 1.00 | — | 1.00 | — | 1.00 | — | 1.00 | — |
| Full | 1.16 | (0.79,1.71) | 1.51 | (0.89,2.55) | **1.34** | **(1.20,1.50)** | 1.10 | (0.94,1.29) |
| Intermediate | 1.41 | (0.85,2.33) | 1.90 | (0.91,3.97) | **1.37** | **(1.18,1.59)** | 1.08 | (0.86,1.36) |
| Limited | 0.51 | (0.21,1.27) | 0.74 | (0.26,2.07) | **1.71** | **(1.45,2.00)** | **1.30** | **(1.02,1.66)** |
| **aOR**: adjusted odds ratios; **aIRR**: adjusted incidence rate ratios.  **^a^** Original models adjusted for age, highest educational level attained, healthcare system used, administrative health region, parity, and complications during pregnancy, n=2407**.**  **^b^** Additionally adjusted for length of stay in Portugal in years (native women’s age was assumed to be their length of stay), n=2402.  Models were based on complete-case analysis. | | | | | | | | |
